# Supplementary material for: A solid-state lithium-ion battery with micron-sized silicon anode operating free from external pressure
Source: Nat Commun. 2024 Mar 13;15:2263. doi: 10.1038/s41467-024-46472-9 (PMC10937906; doi:10.1038/s41467-024-46472-9)
Supplement: Supplementary file 1 — Supplementary Information [file 41467_2024_46472_MOESM1_ESM.pdf]

Supplementary Information

A solid-state lithium-ion battery with micron-sized silicon anode operating free from external pressure

Hui Pan[1], Lei Wang[1], Yu Shi[1], Chuanchao Sheng[1], Sixie Yang[2], Ping He[1*] and Haoshen Zhou[1*]

[1]Center of Energy Storage Materials & Technology, College of Engineering and Applied Sciences, Jiangsu Key Laboratory of Artificial Functional Materials, National Laboratory of Solid State Microstructures and Collaborative Innovation Center of Advanced Microstructures, Nanjing University, Nanjing 210093 (P. R. China)

[2]School of Materials Science and Intelligent Engineering, Nanjing University, Suzhou 215163 (P. R. China)

* E-mail: pinghe@nju.edu.cn; hszhou@nju.edu.cn

Supplementary Note

Typically, N-methylacetamide (NMA) (melting point T_m=35°C) and lithium bisfluorosulfonimide (LiFSI) (T_m=143°C)[1] are solid at ambient temperature, but the mixtures of NMA and LiFSI in different proportions were transparent DEM liquids (Supplementary Fig. 1). Infrared spectroscopy was conducted to study the intermolecular interaction in the DEM (Supplementary Fig. 2a). The peak corresponding to C=O in NMA at 1646 cm^{-1} shifted to 1652 cm^{-1} after mixing with LiFSI due to the coordination between Li^+ and O atom of C=O[2] (Supplementary Fig. 2b). At the same time, the blue shift of peaks corresponding to H-bonded NH I (3286 cm^{-1}) and H-bonded NH II (3098 cm^{-1}) suggested the formation of hydrogen bonds between NH of NMA and O of LiFSI[3]. In addition, Raman peak of LiFSI at 776 cm^{-1} shifted to lower wavenumber after mixing with NMA (Supplementary Fig. 3a),which also indicated the dissociation of lithium salt due to the Lewis acid-base interaction[4]. The dissociation of LiFSI was enhanced as the concentration of lithium salt decreased, leading to a higher ionic conductivity of the DEM (Supplementary Fig. 3b). The ionic conductivity of the DEM reached its maximum when the molar ratio of NMA:LiFSI was 4:1 (Supplementary Fig. 3c). Further reducing the proportion of LiFSI would result in a lower ionic conductivity because of the lower concentration of carrier. Consequently, DEM used in this work hereafter specifically refers to the eutectic mixture of NMA and LiFSI in the molar ratio of 4:1.

Supplementary Figures

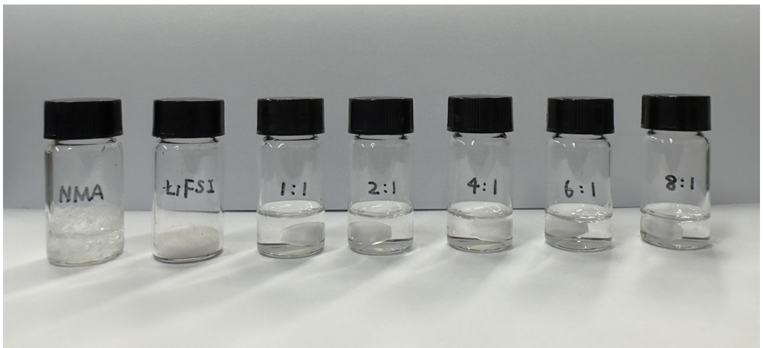

Supplementary Fig. 1. Digital images of NMA, LiFSI and the DEM with different molar ratio of NMA and LiFSI.

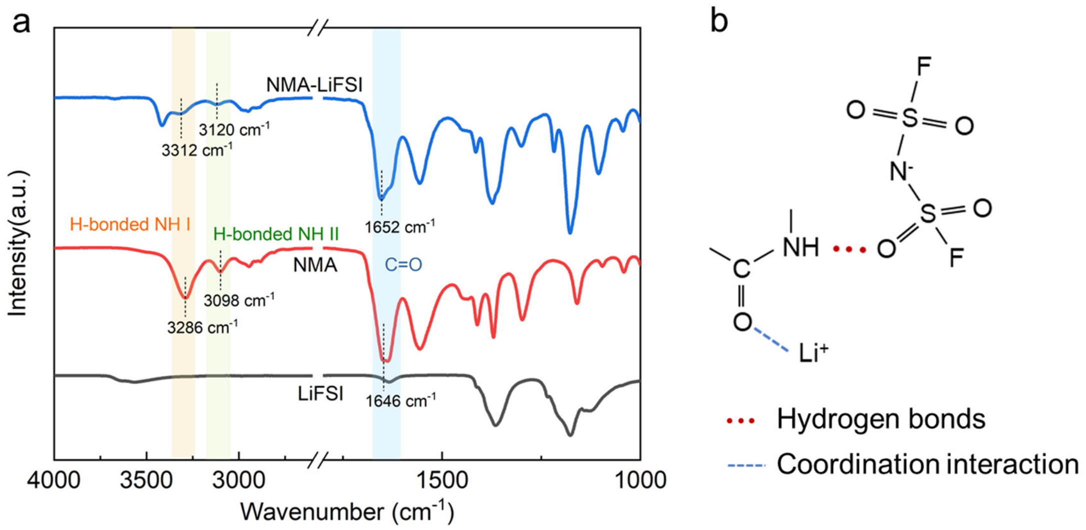

Supplementary Fig. 2. The intermolecular interaction in the DEM. (a) Infrared spectroscopy of NMA, LiFSI and the DEM with molar ratio of NMA:LiFSI=4:1. (b) Schematic of the intermolecular interaction between NMA and LiFSI.

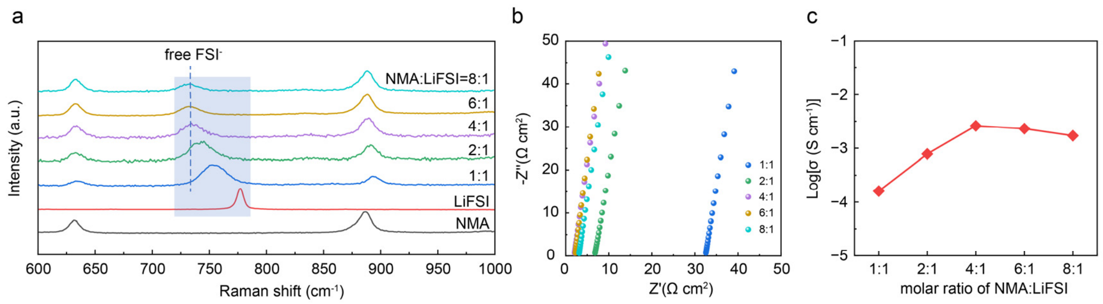

Supplementary Fig. 3. The dissociation of LiFSI in the DMEs and the ionic conductivity of the DEMs. (a) Raman spectra of NMA, LiFSI and the DEMs with different molar ratio of NMA:LiFSI. (b) Nyquist plots and (c) the ionic conductivity of the DEMs with different molar ratio of NMA:LiFSI.

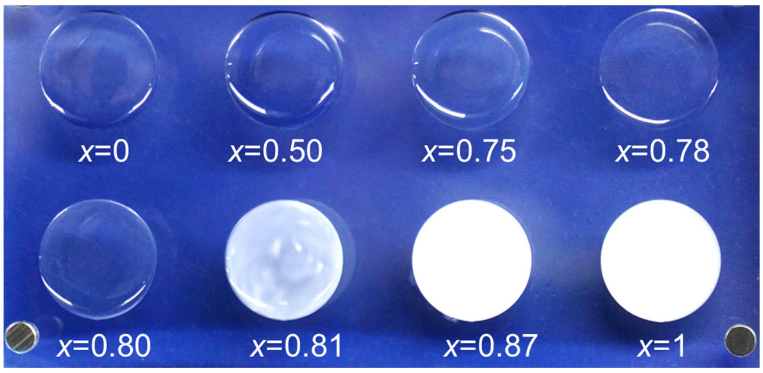

Supplementary Fig. 4. Digital images the copolymer with different ratios of poly-AM, wherein *x* refers to the ratio of poly-AM. The opacity of the copolymer increased with the ratio of poly-AM, with a transition point at *x*=0.8.

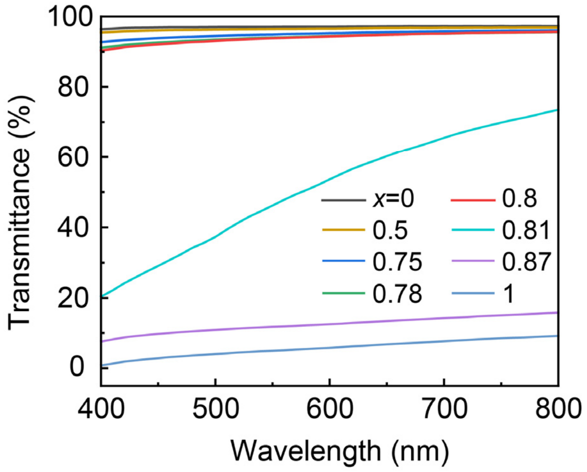

Supplementary Fig. 5. Transmittance of visible light through the elastic electrolyte. The transmittance at 400 nm was higher than 90% when $x \le 0.8$, and it sharply decreased to 20% when $x=0.81$.

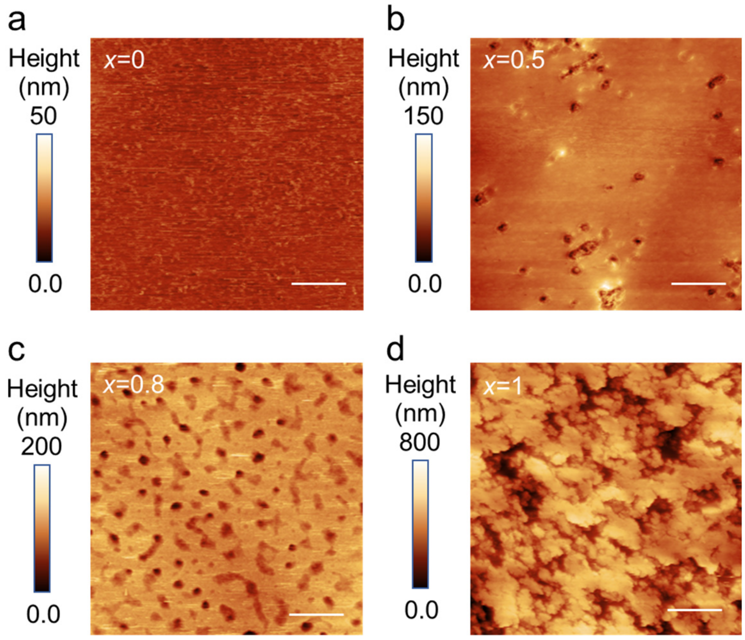

Supplementary Fig. 6. Atomic force microscopy images of the elastic electrolyte with (a) x=0, (b) x=0.5, (c) x=0.8, (d) x=1, wherein x refers to the ratio of poly-AM. The scale bar was 2 μm.

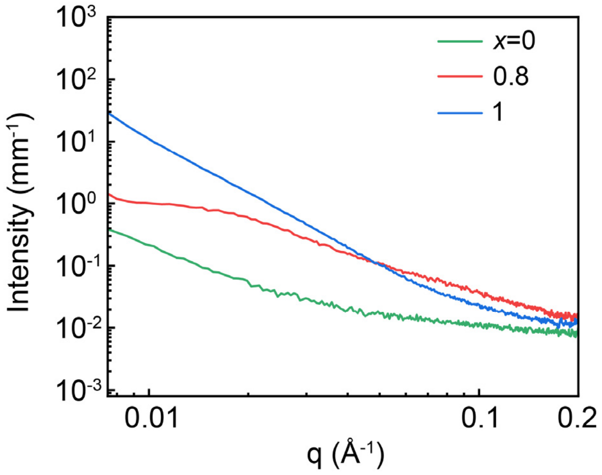

Supplementary Fig. 7. Small-angle X-ray scattering profiles of the electrolyte with different ratios of poly-AM.

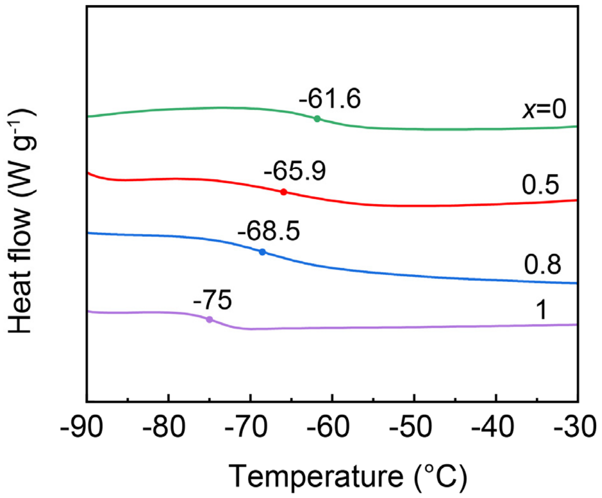

Supplementary Fig. 8. The differential scanning calorimetry curves of the elastic electrolyte with different ratios of poly-AM.

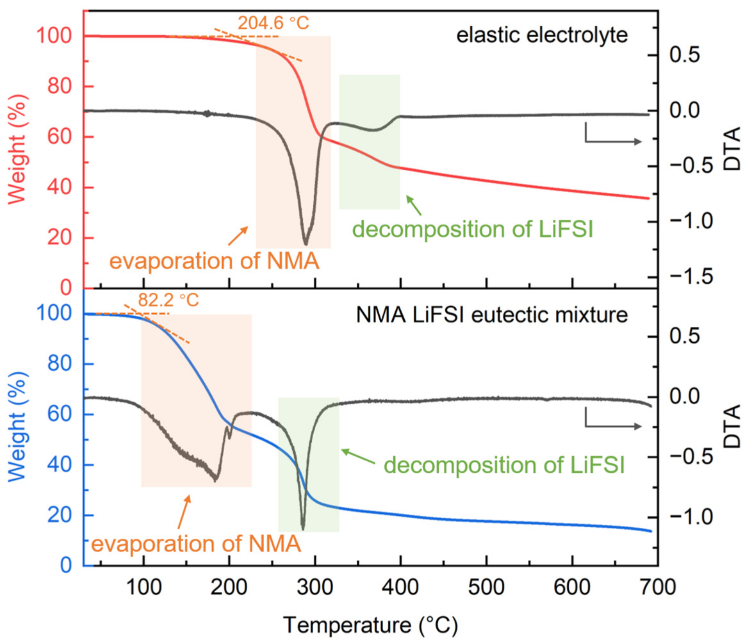

Supplementary Fig. 9. Thermogravimetric analysis curves of the elastic electrolyte and the NMA-LiFSI eutectic mixture.

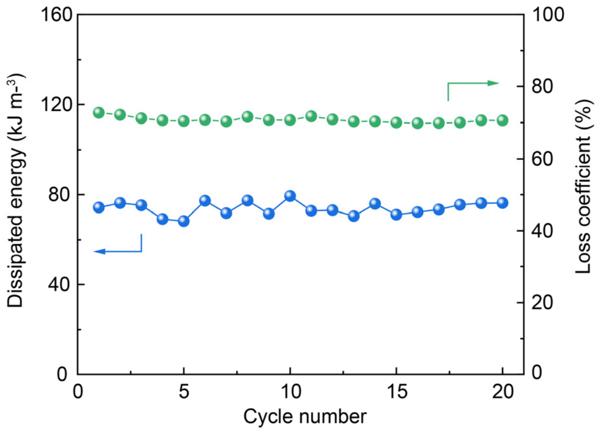

Supplementary Fig. 10. The energy dissipation and loss coefficient of the elastic electrolyte with x=0.8 and the compression ratio of 50%.

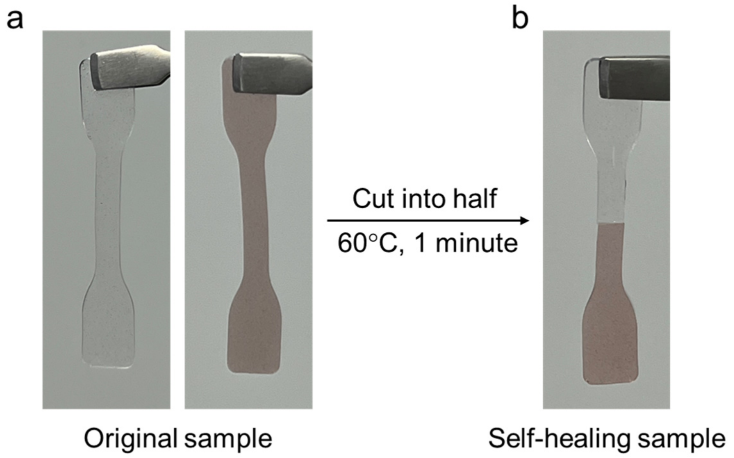

Supplementary Fig. 11. Digital images of (a) the original elastic electrolyte samples with achromatic color (left) and pink color (right) and (b) the self-healing elastic electrolyte sample.

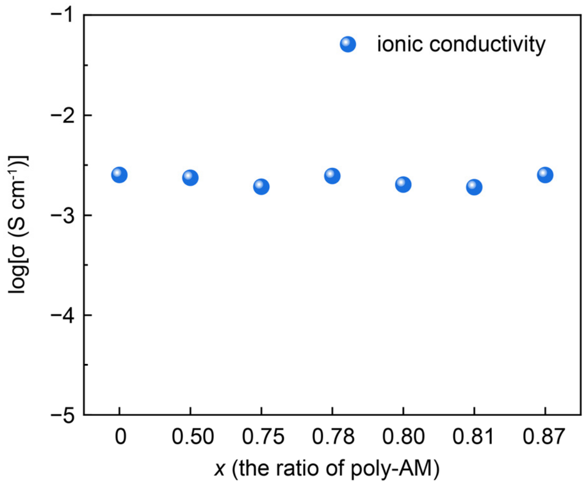

Supplementary Fig. 12. Ionic conductivity of the elastic electrolyte with different ratios of poly-AM.

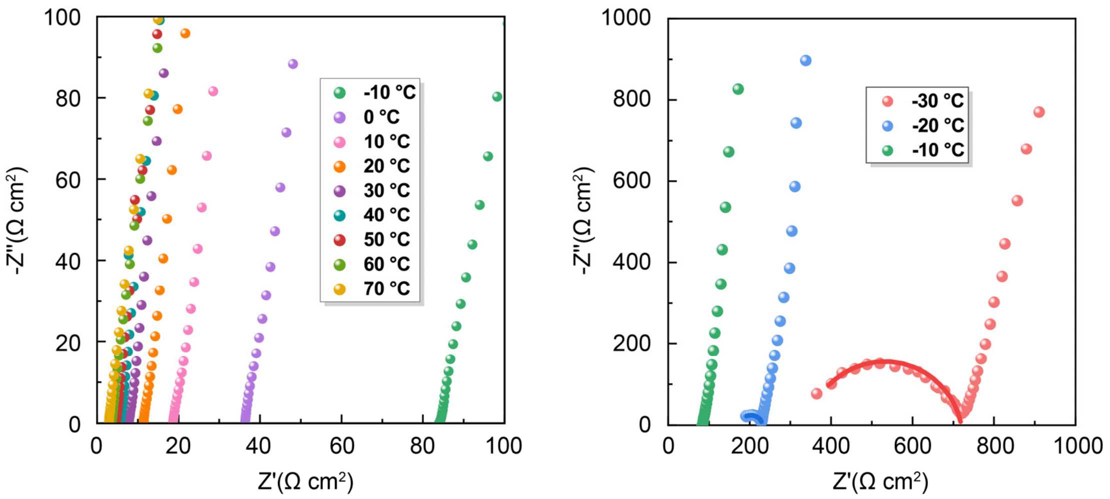

Supplementary Fig. 13. Nyquist plots of the elastic electrolyte at different test temperature.

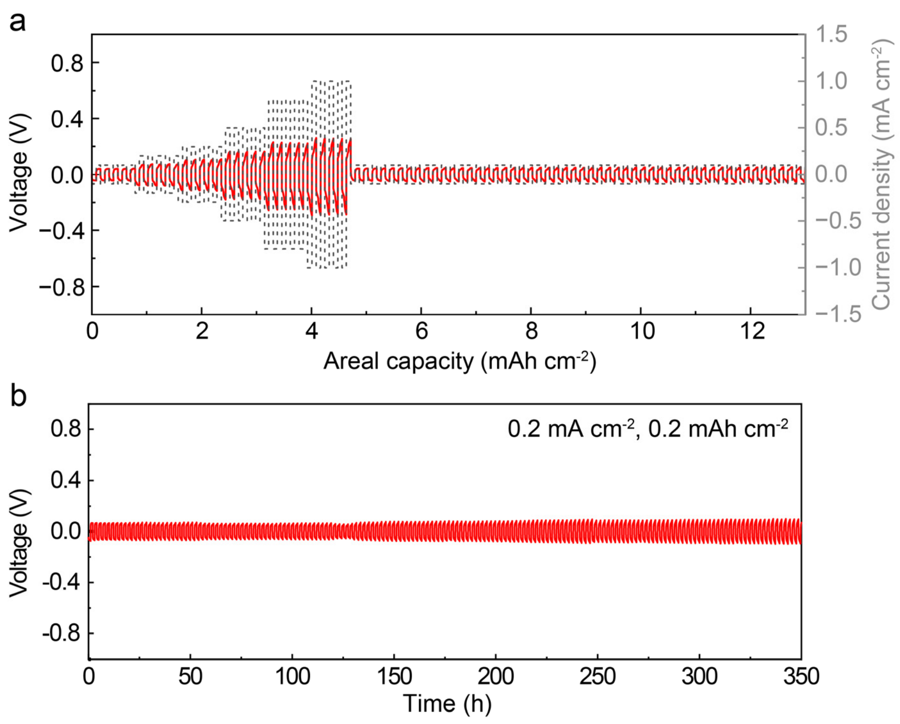

Supplementary Fig. 14. The electrochemical stability of the elastic electrolyte to Li. (a) Rate performance of the Li/elastic electrolyte/Li symmetric cell with an areal capacity of 0.1 mAh cm^{-2}. (b) Galvanostatic Li plating and stripping profiles of the Li/elastic electrolyte/Li symmetric cell at 0.2 mA cm^{-2}, 0.2 mAh cm^{-2}. The symmetric cells were tested without external stack pressure.

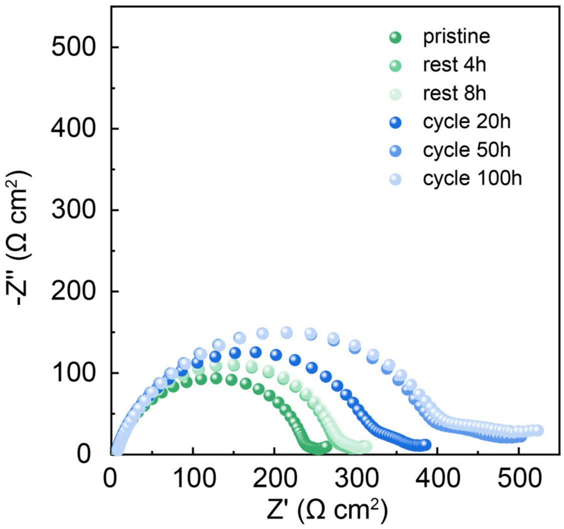

Supplementary Fig. 15. Nyquist plots of the Li/elastic electrolyte/Li symmetric cell during resting and galvanostatic cycling.

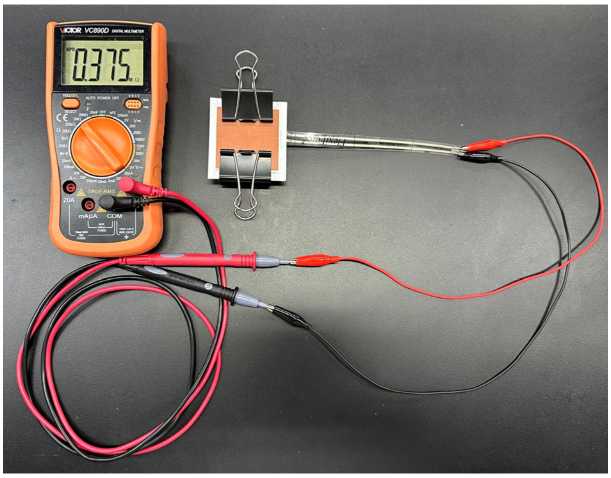

Supplementary Fig. 16. Digital images of the built-in pressure measuring of the pouch-type cell using the membrane force-sensitive resistance (MFSR). The resistance of the MFSR was 0.375 MΩ, corresponding to the internal pressure of 52 kPa in the pouch-type cell. The clamps were used to maintain the electrical contact inside the pouch-type cell.

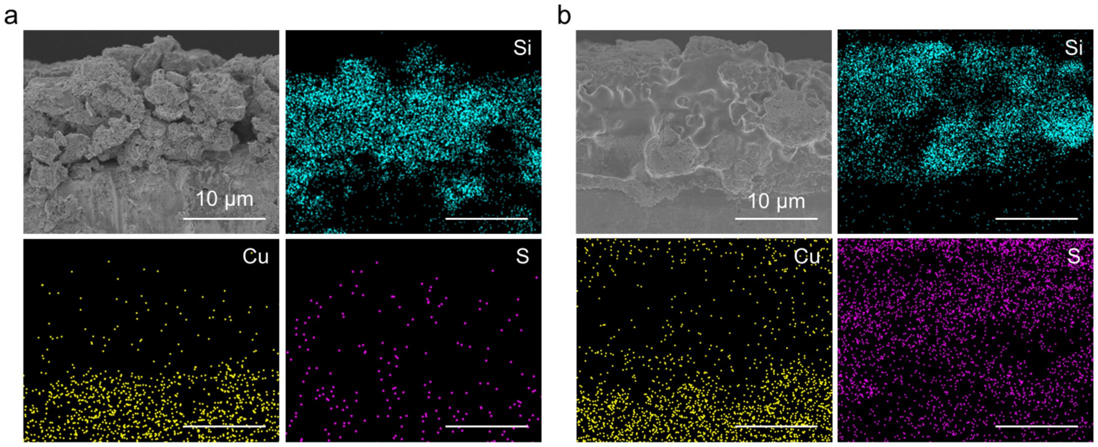

Supplementary Fig. 17. SEM images and the corresponding energy dispersive spectrometer spectra of the µm-Si electrode (a) before and (b) after the infiltration of the elastic electrolyte. The energy dispersive spectrometer spectra of Si, Cu and S is represented in blue, yellow and pink, respectively.

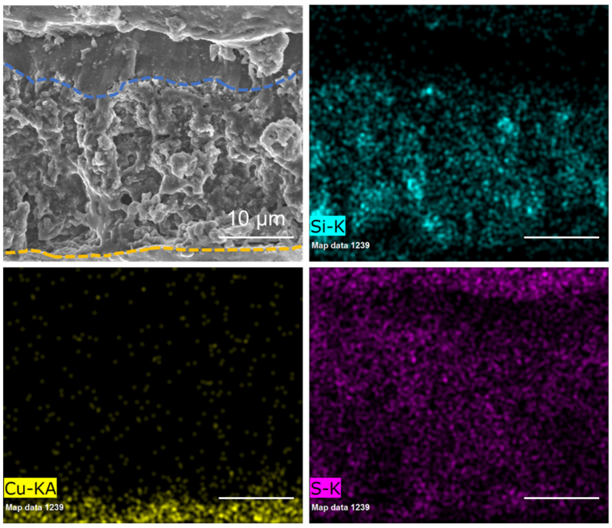

Supplementary Fig. 18. SEM images and the corresponding energy dispersive spectrometer spectra of the μm-Si electrode after cycle in the Li/elastic electrolyte/μm-Si cell. The energy dispersive spectrometer spectra of Si, Cu and S is represented in blue, yellow and pink, respectively.

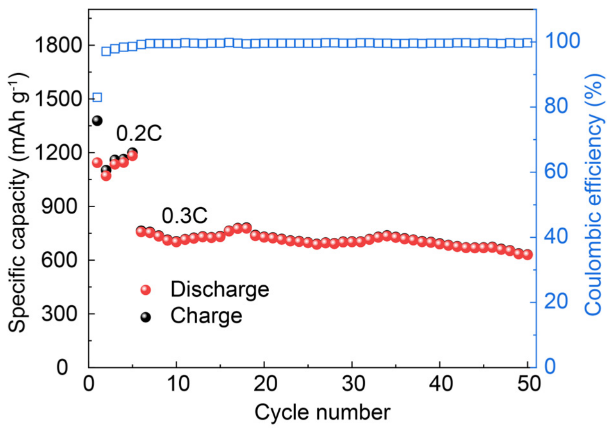

Supplementary Fig. 19. The galvanostatic discharge and charge test on the Li/elastic electrolyte/μm-Si cell with a μm-Si loading of 1.3 mg cm^{-2} at 0.2C and 0.3C without external stack pressure.

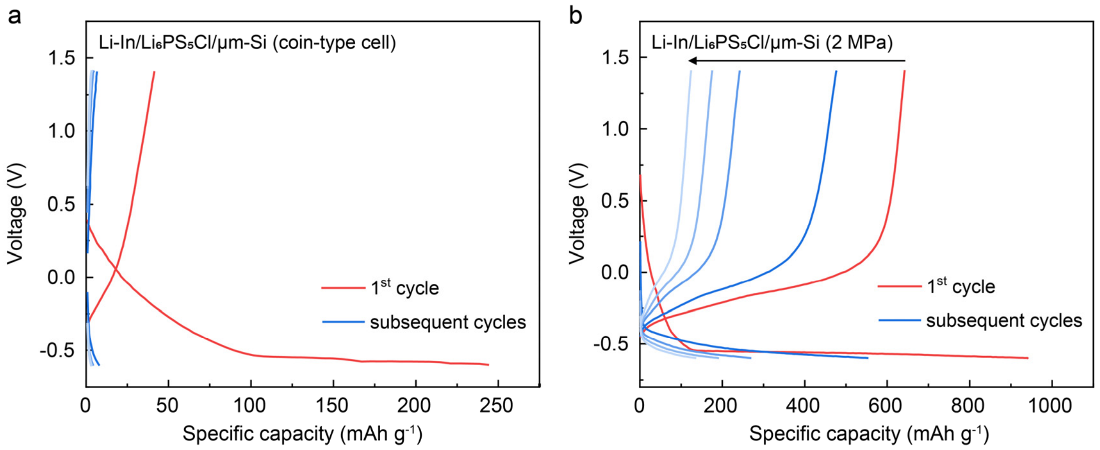

Supplementary Fig. 20. Galvanostatic discharge/charge curves of (a) the coin-type Li-In/Li_6PS_5Cl/μm-Si cell without additional pressure and (b) the Li-In/Li_6PS_5Cl/μm-Si mold cell under a stacking pressure of 2 MPa.

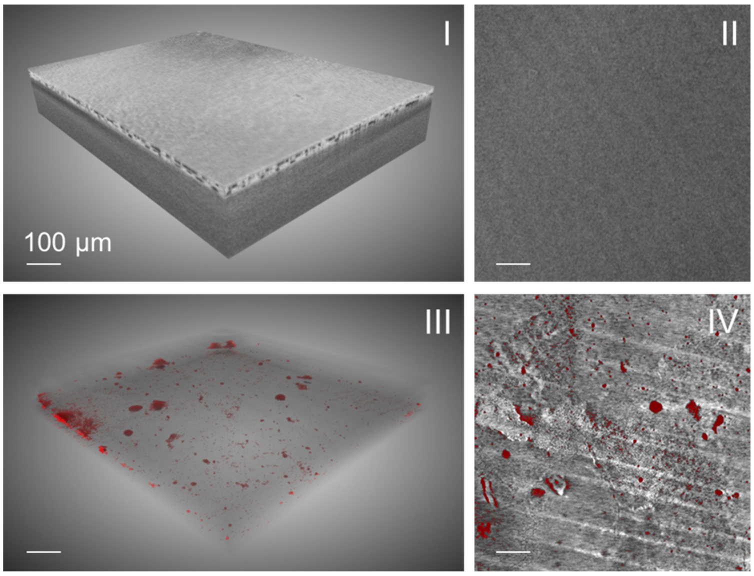

Supplementary Fig. 21. X-ray computed tomography of the pristine µm-Si electrode. Reconstructed 3D structure of the pristine µm-Si electrode with (I)-(II) the elastic electrolyte and (III)-(IV) Li_6PS_5Cl electrolyte. The pores inside the electrode were represented in red. The scale bar was 100 µm.

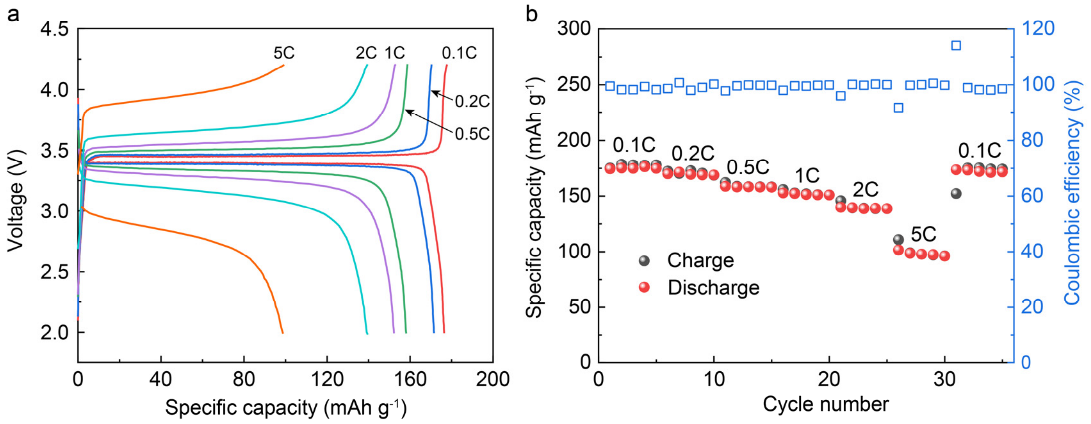

Supplementary Fig. 22. Rate performances of the coin-type Li/elastic electrolyte/LFP battery. (a) The charge-discharge curves and (b) the specific capacity and coulombic efficiency of the cell at different rates.

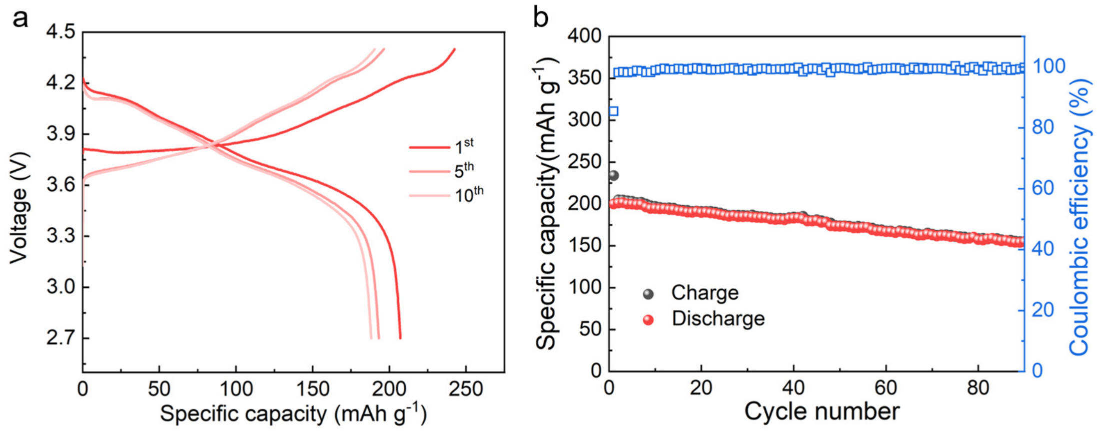

Supplementary Fig. 23. Electrochemical performances of the NMC cathode with the elastic electrolyte. (a) The charge-discharge curve of the 1st, 5th, and 10th cycle and (b) the cycling stability test of the Li/elastic electrolyte/NMC cell with 1 wt% $LiPO_2F_2$ as the additive in the cathode tested without external stack pressure.

Supplementary Table

Supplementary Table 1. The fitting parameters of the Vogel-Tammann-Fulcher (VTF) equation for the elastic electrolyte.

Parameter	A	T_0 (K)	E_a (eV)
Value	1.95±0.59	193.68±9.93	0.0373±0.06

References

1. Amara, S., Zaidi, W., Timperman, L., Nikiforidis, G. & Anouti, M. Amide-based deep eutectic solvents containing LiFSI and NaFSI salts as superionic electrolytes for supercapacitor applications. *The Journal of Chemical Physics* **154**, 164708 (2021).
2. Li, C.L. et al. A Low-Volatile and Durable Deep Eutectic Electrolyte for High-Performance Lithium-Oxygen Battery. *Journal of the American Chemical Society* **144**, 5827-5833 (2022).
3. Chen, Y. et al. The dynamic evaporation process of the deep eutectic solvent LiTf(2)N:N-methylacetamide at ambient temperature. *Physical Chemistry Chemical Physics* **21**, 11810-11821 (2019).
4. Di Pietro, M.E. & Mele, A. Deep eutectics and analogues as electrolytes in batteries. *J Mol Liq* **338** (2021).
