# Supplementary material for: A solid-state lithium-ion battery with micron-sized silicon anode operating free from external pressure
Source: Nat Commun. 2024 Mar 13;15:2263. doi: 10.1038/s41467-024-46472-9 (PMC10937906; doi:10.1038/s41467-024-46472-9)
Supplement: Supplementary file 3 — Description of Additional Supplementary Files [file 41467_2024_46472_MOESM3_ESM.pdf]

Description of Additional Supplementary Files

File Name: Supplementary Movie 1

Legend: A movie showing the stretchability and deformation recovery of the elastic electrolyte. The recovery process was accelerated 10 times.
